# A computational model of the moth macroglomerular complex

**DOI:** 10.1186/1471-2202-12-S1-P212

**Published:** 2011-07-18

**Authors:** Hana Belmabrouk, Jean-Pierre Rospars, Dominique Martinez

**Affiliations:** 1 UMR 7503, CORTEX, CNRS, Vandoeuvre-lès-Nancy, 54600, France; 2 UMR 1272, PISC, INRA,Versailles, 78000, France

## 

**Figure 1:**
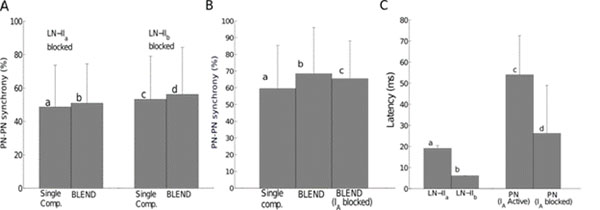
Role of LN inhibition and intrinsic properties on PN synchrony and latency. **A-** Both LN-IIa and LN-IIb are involved in PN-PN synchrony. **B-** Blocking I_A_ current decreases PN-PN synchrony. **C-** Response latencies of LNs (IIa and IIb) and PNs (intact and I_A_ blocked) _._

The macroglomerular complex (MGC) is known as the olfactory sub-system processing pheromonal information. In the moth *Manduca sexta*, the projection neurons (PNs) arborizating in the MGC exhibit two types of responses to pheromone stimulation [1]: a simple monophasic long and tonic excitation (+) and a complex multiphasic pattern (excitation-inhibition (+/-) or inhibition-excitation-inhibition (-/+/-)). The PNs connected to the same glomerulus are synchronized and the level of synchrony is modulated by lateral inhibition [2]. Here we studied the role of inhibition and intrinsic properties of PNs on their synchrony and their response patterns. We developed a computational model of the MGC involving two types of inhibitory local neurons (86 LN-IIa, 68 LN-IIb) connected randomly to 41 excitatory PNs [3]. LN-IIa’s have very dense branching in the MGC and are excited briefly by the pheromone blend, whereas LN-IIb’s possess less dense arborizations and respond by a long-lasting excitation. Single cells are modeled as conductance-based neurons with similar intrinsic currents (Na^+^, K^+^, Ca^2+^, A, SK) and leak current but different parameter values for PNs and LNs. First, we examined the effect of inhibition from LNs. In PN responses, the after-hyperpolarization (AHP) was preserved when the LN-IIa were removed but prior inhibition vanishes so that triphasic patterns (-/+/-) are changed to biphasic (+/-). However, removing LN-IIb made the duration of the AHP phase significantly shorter. The synchrony rate was also disturbed by removing one or another of LNs but loss in synchrony remained more important without LN-IIa (fig.1.A). Then we analyzed the effect of the small conductance calcium-dependent potassium (SK) current on the AHP phase. Blocking the SK channel in the PN model disrupted AHP as found experimentally with local injection of Bicuculline. We also investigated whether the latency of the PN response could be due to the A current: its blocking in the model (experimentally this is done by 4-aminopyridine for example) reduced both PN synchrony (fig.1.B) and latency (fig.1.C). As LNs respond significantly faster than PNs, the onset of the first inhibition in the model coincides with inhibition from LNs. Lateral inhibition then plays the role of a “reset” by eliminating the influence of initial conditions. Taken together, these observations suggest that PN synchrony and firing patterns result from a complex interplay between interglomerular inhibition and neuronal intrinsic properties.
